# Immediate Effect of Baricitinib on Arthritis and Biological Disease-Modifying Antirheumatic Drug-Induced Psoriasis-Like Skin Lesions in Two Patients with Rheumatoid Arthritis

**DOI:** 10.1155/2021/8876847

**Published:** 2021-02-05

**Authors:** Yoshifumi Tada, Nobuyuki Ono, Syuichi Koarada

**Affiliations:** Department of Rheumatology, Faculty of Medicine, Saga University, Saga 849-8501, Japan

## Abstract

Biological disease-modifying antirheumatic drugs (bDMARDs) are very effective for treating rheumatoid arthritis (RA). However, they sometimes induce adverse events such as psoriasis-like skin lesions. We describe psoriasis-like skin lesions that developed simultaneously with an RA flare in patient 1 during treatment with abatacept and in patient 2 soon after starting certolizumab pegol. The skin lesions persisted in patient 2 despite stopping certolizumab. Baricitinib was initiated because of RA flare and resulted in immediate beneficial effects on arthritis as well as skin lesions. The RA went into remission in both patients, and the psoriasis-like skin lesions disappeared within four weeks (patient 1) and three months (patient 2).

## 1. Introduction

The introduction of bDMARDs has remarkably changed the strategy of treating RA. Although they are very effective, side effects can include infection, allergies, and autoimmune phenomenon. The psoriasis and psoriasis-like skin lesions that sometimes develop while under bDMARD therapy are now referred to as paradoxical psoriasis [1, 2]. The estimated incidence of psoriasis in the RA population treated with antitumor necrosis factor (TNF) drugs ranges from 1.04 to 2.31/1000 person-years [2], and these lesions are an important cause of bDMARD discontinuation. Recent investigations have shown that the mechanisms of paradoxical reactions to anti-TNF agents are associated with hyperactivation of type 1 interferons (IFN) [3]. We present two patients with RA who developed psoriasis-like skin lesion as a response to bDMARDs and in whom baricitinib rapidly alleviated the skin lesions as well as the RA.

## 2. Case Presentation

### 2.1. Case 1

**Figure 1:**
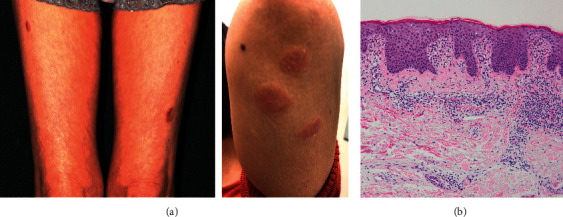
Skin lesion in patient 1. (a) Gross inspection reveals psoriasis-like erythematous lesion on the thighs and left arm. (b) Histological findings show elongated rete ridges, parakeratosis, and mononuclear cell infiltration around dermal vessels (H&E stain ×200).

A 74-year-old woman who developed RA in 2009 had been treated with conventional DMARD, golimumab, and prednisolone for several years, but these became ineffective, and she was referred to our hospital in 2015. She was positive for anticyclic citrullinated peptide antibody (ACPA) (>500 U/mL) and rheumatoid factor (RF, 30 U/L), and X-rays showed erosions in both wrist joints. Treatment with abatacept 500 mg div every four weeks plus methotrexate (MTX) was effective, and the RA went into remission after six months. Prednisolone was then tapered. Two months after MTX withdrawal, in 2018, due to mild liver damage (alanine transferase 114 U/L), the RA flared, and psoriasis-like skin lesions developed in the upper and lower extremities (Figure 1(a)). The patient had no history or family history of psoriasis and never smoked. A skin biopsy showed elongated rete ridges, parakeratosis, and mononuclear cell infiltration around vessels in the dermis (Figure 1(b)) resembling psoriasis. Topical steroids were prescribed by a dermatologist, but the effect was limited. Because her RA had simultaneously become active with the simplified disease activity index (SDAI) of 18.3, we stopped abatacept and started on baricitinib 4 mg/day, which resulted in the SDAI decreasing to 3.54 and the disappearance of the psoriasis-like skin lesions within four weeks. RF also decreased from 423 U/L to 232 U/L in three months. One year later, the RA remained in remission (SDAI: 2.02), and she was free of skin lesions.

### 2.2. Case 2

**Figure 2:**
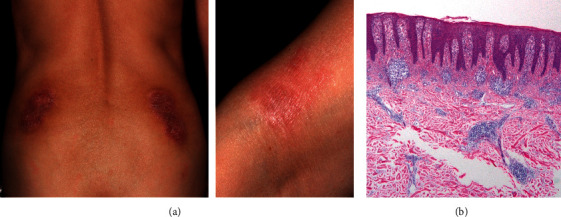
Skin lesion in patient 2. (a) Gross inspection reveals psoriasis-like skin lesions on the back and right elbow. (b) Histological findings show marked elongated rete ridges, mild parakeratosis, and pronounced lymphoid cell infiltration in the upper dermis (H&E stain ×100).

A 65-year-old woman, who developed RA with positive ACPA (>100 U/mL) and RF (27 U/L), was also diagnosed with Sjögren syndrome (anti-SS-A antibody > 500 U/mL). She had been treated with abatacept and golimumab together with MTX, but both bDMARDs were terminated due to the emergence of side effects consisting of liver damage and skin eruptions. Liver biopsy denied autoimmune hepatitis, and liver damage improved with discontinuation of bDMARDs. Two months after starting certolizumab pegol in 2014 (initially, 400 mg s.c. every two weeks for one month followed by 200 mg s.c. every two weeks with MTX at 8 mg/week), skin eruptions appeared on her back and right elbow (Figure 2(a)). A skin biopsy showed marked elongated rete ridges, mild parakeratosis, and pronounced lymphoid cell infiltration in the upper dermis, suggesting psoriasis-like lesions (Figure 2(b)). She had no history or family history of psoriasis and never smoked. After withdrawing certolizumab pegol and topical steroids, the skin lesion in her back gradually disappeared, whereas that in the right elbow persisted. In 2018, we initiated baricitinib (2 mg/day) in addition to MTX (8 mg/week) because of continuous arthritis in her wrist and ankles. This combination exerted a remarkable effect on the RA activity and the skin lesion. SDAI decreased from 8.62 to 3.65 (four weeks) and 1.96 (three months), and the psoriasis-like lesion in the right elbow also disappeared within three months. One year later, the RA remained in remission (SDAI: 1.11), and she was free of skin lesions.

## 3. Discussion

We described two women with RA who developed psoriasis-like skin lesions in response to bDMARD treatment. Baricitinib rapidly resolved the lesions and alleviated the RA in both of them. The pathological and macroscopic findings of the skin lesions were similar between the patients, but the bDMARD and time to onset differed. The lesions appeared after three years of abatacept therapy and, at the same time, as an RA flare in one patient and two months after starting certolizumab pegol in the other. The skin lesions in patient 2 had persisted for almost four years but disappeared under baricitinib within three months. To our knowledge, this effect of baricitinib has not been previously reported.

Anti-TNF agents sometimes induce psoriasis or psoriasis-like skin lesions referred to as paradoxical psoriasis [2], the pathogenesis of which has recently been associated with an increase in type I IFN activity. Increased expression of MxA, a surrogate marker for type I IFN, was initially identified in paradoxical psoriasis induced by TNF inhibitors, suggesting increased production of type I IFN [1]. Recent investigations have found that the immunological phenotypes differ between paradoxical and classical psoriasis; that is, type I IFN activity and plasmacytoid dendritic cell (pDC) infiltration are significantly increased, whereas CD8-T cell infiltration of the dermis is lower in paradoxical psoriasis [3]. A study of a mouse model of skin injury showed that hyperacanthosis, pDC infiltration, and type I IFN expression were enhanced by treatment with an anti-TNF antibody, indicating that a blockade of TNF activity promotes type I IFN overexpression that leads to skin lesions [3]. In support of this theory, it has been shown that TNF-*α* inhibited the generation of pDC from hematopoietic progenitors, and IFN-*α* production from pDC exposed to influenza virus [4]. In addition to paradoxical psoriasis, the adverse immunological effects of anti-TNF agents, antidrug antibody (ADA), and antinuclear antibody (ANA) production are also associated with increased levels of type I IFN, and these can induce secondary unresponsiveness to anti-TNF agents and drug-induced lupus [5, 6]. These findings suggest that anti-TNF therapy sometimes induces an increase in type I IFN activity that can promote paradoxical psoriasis, ADA that leads to a poor response to bDMARDs, and ANA and other autoantibodies that can cause drug-induced lupus.

In addition to anti-TNF drugs, a randomized, placebo-controlled trial showed that abatacept was effective against psoriatic arthritis and psoriatic skin lesions [7], whereas others showed that abatacept induces psoriatic skin lesion as an adverse event [8, 9]. This can be interpreted as another type of paradoxical psoriasis. The psoriasis-like skin lesions and the concomitant RA flare were rapidly alleviated by administering baricitinib in patient 2, suggesting that a common mechanism is involved. The mechanisms remain unknown, but whether type I IFN activity is enhanced in these patients should be investigated.

The treatment of paradoxical psoriasis is challenging. The choice to continue, suspend, or switch the bDMARD has to be carefully made by weighing the severity of the skin lesions against the control of RA. Although bDMARDs can be discontinued in patients with well-controlled RA, active RA should be treated by continuing the same drug or by switching to other types of bDMARDs with different modes of action [10]. In fact, switching to tocilizumab has resulted in good outcomes for both arthritis and anti-TNF-induced skin reactions [11, 12]. Baricitinib is a targeted synthetic antirheumatic drug that mainly inhibits the activities of Janus kinases (Jak) 1 and 2. It inhibits IFN-*α*-induced B-cell differentiation to plasmablasts and IL-6 production by inhibiting Jak signaling [13]. In addition to RA, baricitinib has improved manifestations and IFN biomarkers in patients with various IFN-mediated autoinflammatory diseases, referred to as interferonopathies [14]. It has also proven effective against systemic lupus erythematosus, in which type I IFN are considered to be major players in its pathogenesis [15]. These findings indicate that baricitinib can inhibit enhanced pathological type I IFN activities under various conditions. Considering this background, we treated our patients with baricitinib, which resulted in an immediate response against RA and psoriatic skin lesions.

## 4. Conclusion

Psoriasis-like lesions develop in 2–5% of patients with RA treated by bDMARDs, and its management is a matter of concern, especially when skin lesions persist and/or RA remains active. Because recent investigations have shown that increased type I IFN activity is a significant factor in the development of psoriasis-like skin lesions, Jak inhibitors that block type I IFN signaling might be good candidates for treating this condition. However, more case reports are needed to validate our findings. Furthermore, immunological analyses should provide insight into the mechanisms of baricitinib treatment for paradoxical psoriasis.
